# Executive Decision-Making in the Domestic Sheep

**DOI:** 10.1371/journal.pone.0015752

**Published:** 2011-01-31

**Authors:** A. Jennifer Morton, Laura Avanzo

**Affiliations:** Department of Pharmacology, University of Cambridge, Cambridge, United Kingdom; Université Pierre et Marie Curie, France

## Abstract

Two new large animal models of Huntington's disease (HD) have been developed recently, an old world monkey (macaque) and a sheep. Macaques, with their large brains and complex repertoire of behaviors are the ‘gold-standard’ laboratory animals for testing cognitive function, but there are many practical and ethical issues that must be resolved before HD macaques can be used for pre-clinical research. By contrast, despite their comparable brain size, sheep do not enjoy a reputation for intelligence, and are not used for pre-clinical cognitive testing. Given that cognitive decline is a major therapeutic target in HD, the feasibility of testing cognitive function in sheep must be explored if they are to be considered seriously as models of HD. Here we tested the ability of sheep to perform tests of executive function (discrimination learning, reversal learning and attentional set-shifting). Significantly, we found that not only could sheep perform discrimination learning and reversals, but they could also perform the intradimensional (ID) and extradimensional (ED) set-shifting tasks that are sensitive tests of cognitive dysfunction in humans. Their performance on the ID/ED shifts mirrored that seen in humans and macaques, with significantly more errors to reach criterion in the ED than the ID shift. Thus, sheep can perform ‘executive’ cognitive tasks that are an important part of the primate behavioral repertoire, but which have never been shown previously to exist in any other large animal. Sheep have great potential, not only for use as a large animal model of HD, but also for studying cognitive function and the evolution of complex behaviours in normal animals.

## Introduction

The ability to learn associations between stimuli, actions and outcomes, and to then adapt ongoing behavior to changes in the environment is arguably one of the fundamental determinants of survival. When such ‘executive’ function breaks down (as happens in disorders such as HD, Alzheimer's disease (AD), and schizophrenia) the effect on the individual is devastating, but the distress it causes spreads beyond the affected individual to impact on both families and society. A major effort is underway to develop therapies to halt cognitive decline in neurological disorders. Currently, most preclinical testing is conducted using rodents. While undoubtedly these make useful and economical animal models, they have limitations, particularly when the aim is to test cognitive function in neurodegenerative disorders. Not only are rodents short-lived (which excludes the possibility of studies conducted in a timeframe that is relevant to human disorders), but they also lack some major anatomical characteristics of the human brain, especially the forebrain. For example, rodents do not have a separate caudate and putamen, they do not have distinguishable subdivisions of the globus pallidus, and they do not have a subthalamic nucleus. Rodents also do not have the gyrencephalic cortex that is characteristic of the human brain. These anatomical differences may be particularly important when studying the functions of the brain regions (e.g. basal ganglia and cerebral cortex) involved in complex processes such as motor control and decision-making.

In order to address some of the limitations of rodent models, two new transgenic HD models have been developed, a monkey (Macaca mulatta) [1] and a sheep (Ovis aries) [2]. However, it is immediately apparent that there will be problems using either model for cognitive testing. The HD monkey is a rhesus macaque, a species widely used for studying brain function. Macaques are large monkeys and difficult to manage in a laboratory setting. If the HD monkeys show the profound motor and psychiatric decline that would be expected if the model recapitulates the symptoms of HD, as well as the expected progressive cognitive symptoms, then studying these animals will be particularly challenging. These issues have been alluded to [3], but not yet addressed. It seems unlikely that the monkey model of HD will be widely used for therapeutic testing.

In comparison to monkeys, management of sheep (as farm animals) is routine, and they are widely used in many spheres of basic and pre-clinical research [4]–[8]. However, their cognitive abilities are poorly characterized. Nevertheless, sheep have attributes that should make them suitable for use as animal models for studying cognitive function. They are long-lived, and have large brains with human-like basal ganglia and well-developed, convoluted cerebral cortices (see [2] for references). They also have an impressive ability to remember the faces of other sheep [9], suggesting a good capacity for learning and memory. What is missing is any evidence that sheep would make good experimental subjects for the systematic cognitive testing relevant to neurological disorders. This would be essential if sheep are to make useful models of HD. The aim of our study was to fill this gap.

We focused on two tasks used for testing cognition in patients with neurological disorders; reversal learning and attentional set shifting. Reversal learning is used to test the functional integrity of striatum and pre-frontal cortex in patients [10]–[12]. We reasoned that if normal sheep could perform reversal learning, then this task would be extremely useful for measuring cognitive performance in the HD sheep. The other task we used, attentional set shifting, is a measure of executive function that deteriorates particularly early in HD [13], [14]. Both old world monkeys [15], [16], (see [17] for other references), and new world monkeys [18]–[21] are able to perform attentional set-shifting tasks. Our motivation for including attentional set-shifting in our study was driven more by curiosity than expectation, since it is difficult to train primates to perform this task, and it has been a particularly challenging to establish this test for use in rodents (for references and discussion, see below).

## Materials and Methods

### Animals

Studies were carried out in accordance with the U.K. Animals (Scientific Procedures) Act, 1986. No licensed procedures were carried out in the course of these experiments. We used 7 female Welsh Mountain sheep that were approximately 5 month old when we purchased them, and approximately 1 year old when we started our studies. They were naïve to cognitive testing, and had had no pretraining or handling before they came to us (other than routine farm practice). All but one of the sheep completed the whole study. One sheep broke its leg (accidentally in the field) in the last phase of testing, and so did not complete the last 2 days of the experiment. The sheep lived in a polytunnel enclosed within a small paddock with unlimited access to water. None of the sheep was food-deprived. While they received a supplementary ration of sheep nuts (that were used as the reward), they had ready access to a full ration of hay each day before testing began, and they lived in a paddock where they could graze on grass. On non-testing days, they were given a full ration of sheep nuts.

### Test apparatus

**Figure 1:**
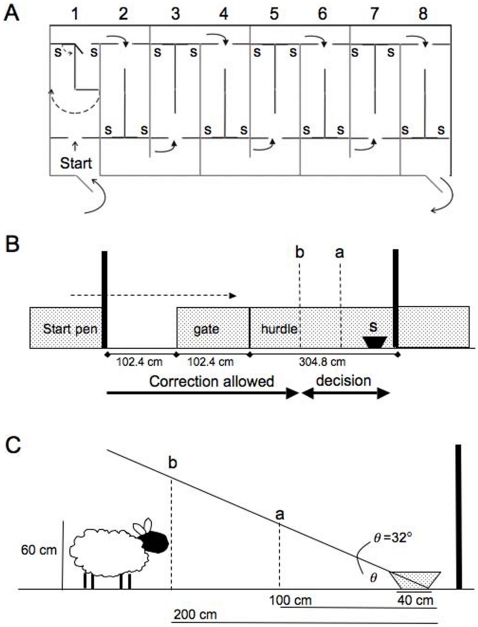
Plan of the testing apparatus. Each of the eight pens (1–8; 2.375 m×9.6 m each) was divided by a sheeted hurdle attached to a gate that could be closed behind the sheep after it chose one of the two stimuli (s) placed at the far end of the pen. The sheep would move out of the start pen (A, B) and move towards the stimuli (dashed arrow). When it reached the gate it would have to choose to go down one or other side of the pen to reach one of the stimuli. The sheep was allowed to self-correct if it turned around before it had reached point b (B, C). Point a is the point beyond which the tallest sheep might be able to see into the bucket.

The test apparatus was a set of 8 outdoor pens (8.5 m×2.5 m). Each pen was divided longitudinally by a metal-sheeted hurdle and attached sheeted gate into a system of runs and gates (Fig. 1).

### Habituation

Animals were handled intermittently (1–2 times per week for up to 30 minutes) for approximately 4 months before formal testing began. In the weeks before testing began, sheep were habituated to the test apparatus on 5 separate occasions for 5–10 minutes each, first in a group, and then individually. During habituation, their normal daily ration of food was distributed between 16 black or green buckets placed at the end of each lane. The sheep were allowed to explore the testing area and to eat any pellets they found in the buckets. None of the gates was closed during the habituation. When the animals exited the test area they were returned to the holding pen.

### Testing paradigm

Testing was conducted using stimuli shown in Table 1, starting with a simple discrimination (SD), that was followed by a simple discrimination reversal (SR), retention trial (Ret), compound discrimination (CD), intradimensional shift (IDS), intradimensional shift reversal (IDR), extradimensional shift (EDS) and extradimensional shift reversal (EDR). For each discrimination, pairs of stimuli, one correct (S+) and one incorrect (S-), were placed on either side of the dividing hurdle at the end of each pen, 6 m from the start gate. The operator, by opening a gate that allowed the sheep into the first start pen, initiated each set of discriminations. Once in the start pen, the animal could see both the S+ and S- in the first pen, and was free to move into the test area towards either of the stimuli. When the sheep had chosen one or other stimulus, for all except the first 8 sets of discriminations on each new major paradigm, the holding gate was closed behind it.

**Table 1:** Order of Discriminations.

Discriminations	Dimension		Exemplar combinations			
	Relevant	Irrelevant	Correct	Irrelevant	Incorrect	Irrelevant
Simple discrimination (SD)	Colour	-	C1		C2	
Retention (Ret 1)	Colour	-	C1		C2	
Simple reversal (SR)	Colour	-	C2		C1	
Retention (Ret 2)	Colour	Second bucket	C2	Second bucket	C1	Second bucket
Compound discrimination (CD)	Colour	Shape	C2	S1	C1	S1
Intradimensional shift (IDS)	Colour	Shape	C3	S1, S2	C4	S1, S2
Intradimensional reversal (IDR)	Colour	Shape	C4	S1, S2	C3	S1, S2
Extradimensional shift (EDS)	Shape	Colour	S1	C3, C4	S2	C3, C4
Extradimensional reversal (EDR)	Shape	Colour	S2	C3, C4	S1	C3, C4

C1 =  blue, C2 =  yellow, C3 =  purple, C4 =  green, S1 =  cone, S2 =  trapezoid.

For the first 8 discriminations in SD, SR, ID, IDR, ED, EDR, if an animal made an incorrect choice, it was allowed to make a correction, whereby it could return to the other lane and collect the reward. For all other discriminations, once the choice had been made, a gate was closed behind the sheep, enclosing it in a smaller area with the S+ or S-.

Reinforcement was a portion (3–5 pellets) of the sheep's normal daily ration of feed. If the choice was made correctly, a sheep was allowed to eat the pellets. When it had eaten the pellets, a second gate was opened that allowed the sheep to proceed to the next set of pens. If the choice was incorrect, the sheep had to wait for 20 s before being allowed through the second gate to the next set of pens. ‘Correct’ or ‘incorrect’ choice was assigned once the sheep had passed a defined point in the test area (**b** in Fig. 1B, C). This point was defined as 2× distance from the back of the bucket to point **a**, where **a** was the point at which the tallest sheep might be able to see pellets in the bottom of the bucket. We were confident that the sheep were using visual rather than olfactory cues to locate the pellets, because during training, if we used buckets with pale brown inserts (that were the same colour as the pellets) the sheep could not find the pellets by smell alone. At the end of the testing session, the sheep were returned to their home paddock and given the remainder of their rations.

Testing was conducted on 21 days between March and June 2010. All sheep performed the same series of discriminations (Table 1). In all parts of the test, the food reward was placed in the bottom of a feed bucket. For the SD and SR, two buckets, identical except for colour (yellow or blue) were used, and the bucket that was the S+ contained the reward. For the CD, the relevant dimension (colour) remained unchanged, but the blue/yellow buckets were swapped for blue/yellow plastic perforated sports cones. An additional bucket (either black or green) was placed adjacent to each S+ and S-, with the one next to the S+ containing the reward. For the CD (that followed the SR) the S+ was blue. From then on, for all discriminations, the S+ and S- were objects, and a bucket was placed next to each S+ and S-; the bucket next to the S+ contained the food reward.

For the IDS and EDS, the dimensions used were colour and shape respectively. The objects used as the S+/S- were either a cone or an inverted bucket (rhomboid) wrapped in a piece of sheeting material cut from a single piece of similarly shaded coloured cloth (purple or green). For the IDS, the choice of stimuli changed from yellow/blue to purple/green. The pairs of exemplars were always equally represented within groups, as was the location (left or right) of the S+. The order of exemplars used, and the side-of-stimulus presentation was determined by an *a priori* pseudorandom list. For the EDS and IDS, 3 of the sheep were trained to one colour (IDS) or shape (EDS) respectively, the remaining 4 the sheep were trained to the other. We did not attempt to counterbalance colour and shape.

### Number of discriminations

In the SD, we conducted only one set of 8 discriminations each per day. This was in part dictated by the weather, which limited our testing, but also in part because we did not want to keep the sheep isolated from their flock mates, given the evidence that isolation in sheep is stressful. However, by the time of the first retention trial (eighth day of testing), it became clear that the sheep would do more than 8 discriminations each day without difficulty. When we increased the number of discriminations from one set of 8 per day to 4–6 sets per day, all of the sheep completed all discriminations without difficulty. Thus, from the SR onwards, 4–6 sets of 8 discriminations were conducted daily. Note that each day, all sheep performed the same number of discriminations; the number of sets of discriminations conducted each day varied only because of the weather. (The pens were outdoors, and testing could not be conducted under windy conditions or in the rain.)

### Data analysis

Choices made, and time-to-choice were recorded for all discriminations of all animals. Other behaviors (pacing, circling, nibbling weeds (defined as ‘displacement activity’), interactions with the objects or buckets (‘irritability’), pawing, bleating, defecation, urination (‘anxiety’) or leaving the test pen to interact with the operator (‘checking’) were also recorded for all discriminations. For the SD and retention (when only one set of discriminations was performed each day) criterion was set at performance of 80% on two consecutive sets (16 discriminations). For SR and all other subsequent components of the testing, criterion was set at 6 consecutively correct choices [15]. All animals completed the same number of discriminations.

Significant differences were assessed using unpaired Student's t-test or by one- or two-way analysis of variance (ANOVA) with Newman Keuls or Duncan's post hoc test, where applicable.

## Results

### Discrimination learning, retention and reversal learning

**Figure 2:**
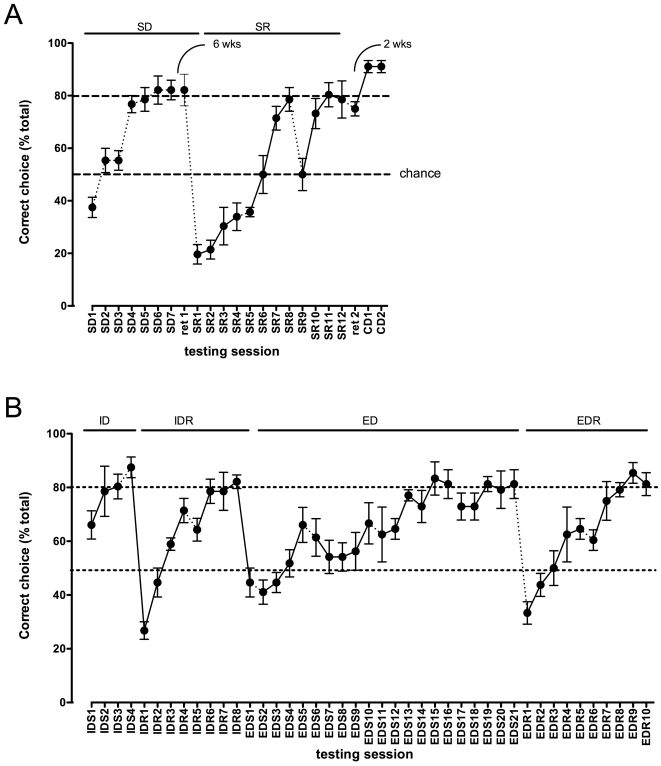
Performance of sheep in the two choice discrimination task. Each point represents the mean (± SEM) number of correct choices made in each set of 8 discriminations. Where points are joined by solid lines, the sets of discriminations were tested on the same day. Where points are joined by dotted lines, testing was conducted on a different day. SD =  simple discrimination, SR  =  simple discrimination reversal, Ret1 =  first retention trial, CD =  compound discrimination IDS = intradimensional shift, IDR  =  intradimensional shift reversal, EDS =  extradimensional shift, EDR = extradimensional shift reversal.

All of the sheep learned to discriminate between coloured (yellow and blue) buckets, reaching criterion in the simple discrimination (SD) within 7 sets of 8 discriminations (Fig. 2A). Sheep were re-tested on the task 6 weeks later (Ret 1), and reached criterion within 1 set of discriminations. Thus, they could remember the correct choice for at least 6 weeks.

**Figure 3:**
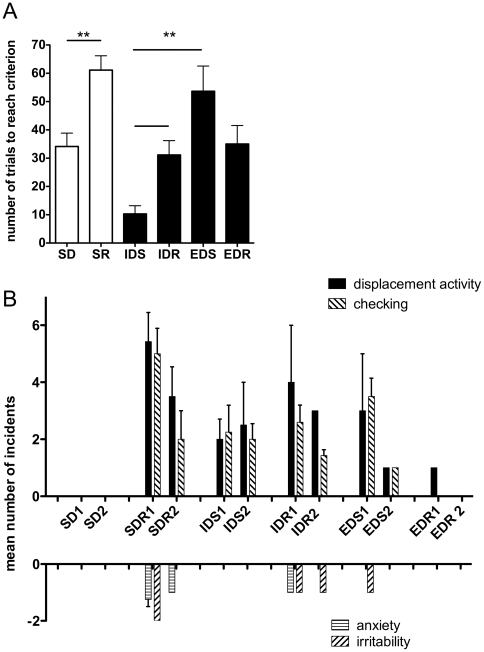
Comparison of number of trials to reach criterion in different stages of the task (A) and number of incidences of ‘emotional’ display (B). Abbreviations for different phases of the trials are described in Fig. 2.

When the stimulus-reward contingency was reversed (simple reversal; SR) so that the previously correct stimulus was now incorrect, there was a pronounced decrease in correct choices as the sheep continued to choose the previously correct S+ (SR1, Figure 2A). Nevertheless, the sheep learned the reversal, and reached criterion after 3 days of testing (11 sets of discriminations). All of the sheep required significantly more discriminations to reach criterion for SR than for the SD (Fig. 3A; p<0.01). Interestingly, the behavior of the sheep during SR suggested that not only had they learned that the S+ was correct, but they had also learned that the S- was incorrect. Although the only ‘punishment’ they received for an incorrect choice was to wait for 20 s (and no reward), their behavior when the rule was changed was striking. In the first set of 8 discriminations for both the SD and the SR, the sheep were allowed to self-correct. During SD, if they chose incorrectly, they immediately turned and ran to the other lane, to check out the other bucket and collect the reward. However, in the SR, when they found that there was no reward for what had previously been the correct choice, rather than run into the other lane and collect the pellets from the other bucket, they engaged in a number of behaviors that we had not seen hitherto (Figure 3), including perseveration on the previously correct S+, running back to the investigator, pawing the investigator, eating weeds or grass growing in the cracks of the pens, defecating, urinating and bleating. None of the sheep went immediately to the new S+. Indeed, all of the sheep were extremely reluctant to enter the previously incorrect lane. When they eventually did enter the other lane, they did so very slowly, showed behaviors that we had not observed previously (circling, nibbling at weeds), and finally approached the bucket obliquely, rather than taking a direct line to the bucket. These behaviors disappeared as the sheep learned the reversal (Figure 3B).

When the animals had reached criterion on the reversal, compound discrimination was tested using the blue bucket as the S+. Performance of the animals dropped slightly, but within 2 days they were back at criterion (data not shown). When retention was tested 2 weeks after SR12 the mean correct response for the group was 75% (ret 2). We wanted to test whether or not the sheep were using colour to discriminate the S+ or S-. For this, we replaced the blue/yellow buckets with novel blue/yellow objects (perforated football practice cones) as the S+ and S- (respectively). A black or green bucket was placed adjacent to the S+ and S-, and the reward placed in the bucket next to the S+. With a new blue/yellow shape the sheep all performed above criterion, suggesting that they were using wavelength to discriminate between the objects (CD1, CD2; Figure 2A).

### Attentional set shifting

Attentional set shifting was tested over 9 consecutive days. An ID shift (IDS) occurs when a subject trained to respond to a particular stimulus dimension, such as colour or shape, is required to transfer that rule to a novel set of exemplars of that same stimulus dimension. An ED shift (EDS) occurs when a subject is required to shift response set to an alternative, previously irrelevant dimension. We used a novel set of stimuli that were purple or green cones or rhomboids. Colour was the reinforced dimension for the IDS. Mean performance on the first set of discriminations with the new stimuli was not different from chance (66±5% correct), after which performance improved significantly to >90% correct, showing that all of the sheep could discriminate between the new colours. When the discrimination was reversed (IDR1; Figure 2B **)** performance dropped to 27±3% correct (P<0.0001). This improved rapidly and the sheep learned the reversal within 8 sets of 8 discriminations (IDR, Figure 2B). For the EDS, the reinforced dimension was shape (cone or rhombus). On the first set of discriminations of the EDS, performance of all of the sheep dropped from 82±2% to 45±5% correct (p<0.0001). Over the next few days of testing, performance improved very slowly but the sheep learned the task (P<0.0001; repeated measures ANOVA). The group of sheep reached 80% correct on the fourth day of testing. When the EDR was tested, performance dropped significantly on the first set of discriminations, showing that the EDS had been learned (P<0.0001). However, within 2 days (10 sets of discriminations), all the animals had learned the reversal.

Significantly more trials were needed to reach criterion in the reversal (SR and IDR) than in the acquisition of the SD and IDS (Figure 3A). Note however that the reversals in this study are not equivalent, and therefore not directly comparable with each other. Animals were trained slowly on the SD, with only one set of discriminations per day, and testing was spread over several weeks. Retention was then tested on the S+ six weeks later. By contrast, the IDS/IDR and the EDS/EDR were tested in a comparable fashion, on sequential days with multiple sets of discriminations each day and so can be compared directly. The number of discriminations taken to reach criterion in the IDS was significantly fewer than for the EDS (P<0.001). The number of reversals for the IDR and EDR was similar, but because we did not use a total shift paradigm [22], the significance of this is not clear.

### Emotional reactivity during testing

On the first set of discriminations in the SR, the sheep showed significant amounts of displacement activity (Fig. 3B, upper segment of the graph). They also showed novel negative emotional behaviors that had not been seen previously, suggested anxiety/distress relating to the rule change. With subsequent switches in the rule, displacement activity lessened (Figure 3B). The SR, when the first rule change occurred, was the only phase of the testing in which anxiety-like behaviors were seen. The only other cluster of distinctive behavior was observed during the EDS, where if the animals made incorrect choices, they showed displacement activity and irritability. However, they did not exhibit any signs of anxiety.

Although we did not quantify it, positive emotion (ears forward, eye contact with the handler, nuzzling of the handler) was evident in all of the sheep, particularly before each test run began. Sheep showed no reluctance to participate in the testing at any stage.

## Discussion

The first step towards a large animal model of HD has been taken with the development of two new transgenic animals, a non-human primate and a transgenic sheep. Here we investigated the potential for using sheep for systematic cognitive testing. We show that not only can normal can sheep perform discrimination reversal learning tasks, but they can also perform attentional set shifting tasks that test executive function. Thus, quantification of cognitive dysfunction in the sheep model of HD is going to be both possible and practicable. The ability of sheep to perform ID/ED shifts is particularly interesting, because this paradigm has been used successfully to detect basal ganglia and prefrontal function and impairment [15], [20], [21], [23]–[28]. To our knowledge, this is the first time that these executive functions have been demonstrated in any large animal, apart from primates.

Attentional set shifting is a test of rule acquisition and reversal that is a measure of executive function [22], [29]. We were somewhat surprised by the ability of the sheep to perform the attentional set shifting task, since it is a particularly challenging test of cognitive function. Both old world monkeys [15]–[17], and new world monkeys [18]–[21] are able to perform attentional set-shifting tasks. However, this task has been particularly difficult to establish in rodents. While mice can perform SD and SR in the touchscreen [30], we failed to get the ID/ED shift task working in mice using visual stimuli in the touchscreen system. Although one other group has reported some success with this task in mice [31], no difference was found between the performance of the ID and ED shift in mice, in contrast to what is seen in humans and monkeys. Better results have been obtained using textures and odors as the dimensions for measuring set-shifting in rats [25], [32]–[35] and mice [36], [37] although differences in ID/ED shifts are not always seen [35], [36]. At present, the mechanisms underlying attentional set shifting are not fully understood, nor are the species differences. The absence of an ID/ED difference in rodents has been interpreted to mean that mice [31], [36] and, under some circumstances, rats [35] are unable to form perceptual sets (although this may be more a reflection of the difficulties inherent in designing experiments to test this accurately, than a lack of appropriate physiology). It has been suggested previously that rodents and non-human primates may use different strategies for learning this task; for discussion, see [38]. The fact that the sheep showed a significant difference in the number of errors to reach criterion in ID and ED shifts suggests that the strategies used by sheep for these solving tasks may be more similar to humans and non-human primates than to rodents.

The ability of sheep to perform reversal learning and attentional set shifting raises the possibility that they might be useful for testing cognition, not only in models of HD and other diseases in which attentional set shifting is abnormal (e.g. schizophrenia [39]–[42], AD [43] and Parkinson's disease [44]–[47]), but also in normal animals. It is clear from MRI and anatomical studies in humans that the striatum and prefrontal cortex govern both of these behaviors, and that sorting of concept formation (as is required for ID/ED shifts) is particularly sensitive to frontal lobe damage [10], [48]–[49]. Although it has not been shown formally that sheep have the equivalent to the human prefrontal cortex, both mice and rats have equivalent brain regions [50], so there is no reason to think that this would not also be the case for sheep, particularly since they can perform tasks requiring this brain region.

Cognitive testing in sheep need not be restricted to the tasks we have described. There is already evidence that other disease-relevant cognitive behaviors could be tested in sheep, particularly those relating to learning and memory. For example, abnormalities in spatial memory could be tested. Although formal maze testing has only occasionally been conducted in sheep [51], many breeds of sheep can be hefted, suggesting that they have excellent capacity for spatial learning and memory. ["Hefted" means that the sheep have lived a small local area (heft) throughout their lives. Each ewe remains on her heft without the need for fences. Lambs learn their heft from their mothers. They are brought in, only for lambing, dipping and shearing, after which they return to their own part of the mountain instinctively.] Sheep also have good memories for faces [9]. The impressive cognitive abilities of sheep, as well as their ability to discriminate colour and shape (that are used for testing humans, but cannot be used for mice or rats), gives them significant advantages over rodents as experimental animals for testing higher cognitive function.

The amenability of the sheep to training and testing also gives them some advantages over primates. While non-human primates have been used very successfully to study multiple aspects of cognitive behavior (see [52] for references), the challenges associated with doing primate studies, both practical and ethical, mean that fewer and fewer laboratories are now carrying out such studies. Sheep have an agreeable disposition, and make willing [although somewhat rumbustious] experimental subjects. We show that they can be tested individually for at least 30 minutes without showing any sign of distress, which again was something of a surprise, given that sheep in isolation become stressed [53], see [54] for other references. Further, experimental time in sheep was markedly shorter than has been reported in monkeys, where training and testing typically takes many months [17], [21]. By contrast, this whole experiment was completed with only 21 days of testing. Finally, sheep can show both positive and negative emotion (this study, [55]; see [54], [56] for other references. Abnormalities in emotional processing are common in human neurological diseases, but have been refractory to study in rats and mice, and are difficult to study in non-human primates. Our study opens new possibilities for the study of complex emotional as well as cognitive behaviours, not only in the context of neurological disorders, but also in normal animals.
